# Regulation of Wnt Singaling Pathway by Poly (ADP-Ribose) Glycohydrolase (PARG) Silencing Suppresses Lung Cancer in Mice Induced by Benzo(a)pyrene Inhalation Exposure

**DOI:** 10.3389/fphar.2019.00338

**Published:** 2019-05-03

**Authors:** Wenjuan Dai, Yingbin Fu, Yanxia Deng, Zhuoying Zeng, Pan Gu, Hailong Liu, Jianjun Liu, Xinyun Xu, Desheng Wu, Xianru Luo, Linqing Yang, Jinzhou Zhang, Kai Lin, Gonghua Hu, Haiyan Huang

**Affiliations:** ^1^Shenzhen Center for Disease Control and Prevention, Shenzhen, China; ^2^Jiangxi Provincial Key Laboratory of Preventive Medicine, School of Public Health, Nanchang University, Nanchang, China; ^3^Department of Preventive Medicine, Gannan Medical University, Ganzhou, China

**Keywords:** benzo(a)pyrene, ADP-ribosylation, poly (ADP-ribose) glycohydrolase, Wnt signaling pathway, lung cancer

## Abstract

Benzo(a)pyrene (BaP) is a polycyclic aromatic hydrocarbon that specifically causes cancer and is widely distributed in the environment. Poly (ADP-ribosylation), as a key post-translational modification in BaP-induced carcinogenesis, is mainly catalyzed by poly (ADP-ribose) glycohydrolase (PARG) in eukaryotic organisms. Previously, it is found that PARG silencing can counteract BaP-induced carcinogenesis *in vitro*, but the mechanism remained unclear. In this study, we further examined this process *in vivo* by using heterozygous PARG knockout mice (PARG^+/−^). Wild-type and PARG^+/−^ mice were individually treated with 0 or 10 μg/m^3^ BaP for 90 or 180 days by dynamic inhalation exposure. Pathological analysis of lung tissues showed that, with extended exposure time, carcinogenesis and injury in the lungs of WT mice was progressively worse; however, the injury was minimal and carcinogenesis was not detected in the lungs of PARG^+/−^ mice. These results indicate that PARG gene silencing protects mice against lung cancer induced by BaP inhalation exposure. Furthermore, as the exposure time was extended, the protein phosphorylation level was down-regulated in WT mice, but up-regulated in PARG^+/−^ mice. The relative expression of Wnt2b and Wnt5b mRNA in WT mice were significantly higher than those in the control group, but there was no significant difference in PARG^+/−^ mice. Meanwhile, the relative expression of Wnt2b and Wnt5b proteins, as assessed by immunohistochemistry and Western blot analysis, was significantly up-regulated by BaP in WT mice; while in PARG^+/−^ mice it was not statistically affected. Our work provides initial evidence that PARG silencing suppresses BaP induced lung cancer and stabilizes the expression of Wnt ligands, PARG gene and Wnt ligands may provide new options for the diagnosis and treatment of lung cancer.

## Introduction

Benzo(a)pyrene (BaP) is a polycyclic aromatic hydrocarbon that is known to be carcinogenic. It is mainly produced by pyrolysis and incomplete combustion of carbonaceous materials and is widely distributed in both the working and living environment (Liu et al., 2008). A large number of experiments have shown that BaP can induce cancer in various animals (IARC Working Group on the Evaluation of Carcinogenic Risks to Humans, 2010; Kasala et al., 2016). Furthermore, epidemiological studies suggest that BaP is closely associated with human lung cancer (Rojas et al., 2004; Alexandrov et al., 2010; Widziewicz et al., 2018). On the basis of these studies, BaP was classified as a human class I carcinogen by the International Agency for Research on Cancer in 2006 (IARC Working Group on the Evaluation of Carcinogenic Risks to Humans, 2012).

Lung cancer is the most common malignant tumor in the human respiratory system and is extremely harmful to human health. Globally, the morbidity and mortality of lung cancer are among the highest (Ferlay et al., 2015). According to the American Cancer Society, lung cancer leads to the highest number of deaths in both men and women. Recent evidence suggests that the incidence of lung cancer in China is the highest and the mortality is increasing at a rate of 4.5% per year (Chen et al., 2016).

The occurrence of lung cancer is the result of a combination of both environmental and genetic factors, including epigenetic changes which have been proved to contribute to lung cancer development (Hagood, 2014). ADP-ribosylation, as an epigenetic modification, plays a critical role in cell survival and disease development, including cancers (D’Amours et al., 1999; Min et al., 2010; Huang et al., 2012). Poly-ADP-ribosylation can convert nuclear chromatin to a loose state, allowing accessibility of DNA damage repair enzymes to the injury site, thereby promoting DNA damage repair against cytotoxicity and genetic damage. Poly-ADP-ribose glycohydrolase (PARG) can hydrolyze poly (ADP-ribose) on poly (ADP-ribose) polymerase-1 (PARP-1), which promotes the degradation of intracellular poly (ADP-ribose) (PAR) (Rouleau et al., 2004). It is the only known enzyme that can hydrolyze poly (ADP-ribose) in the nucleus (Meyer et al., 2007). Recent studies have shown that PARG gene silencing can increase intracellular poly-ADP-ribosylation to protect cells against cytotoxicity. Li et al. (Li et al., 2016) found that BaP can induce chromosomal aberrations, micronucleus formation, chromatin structure changes and malignant transformation of normal 16HBE cells, but PARG gene silencing can inhibit these abnormalities. Studies have shown that PARG also is associated with tumorigenesis (Miwa and Masutani, 2007), but the exact mechanism of PARG on tumor promotion has not been fully clarified.

In our previous study, 16HBE cells and PARG-deficient cells were treated with 40 μmol/L BaP for a period of time to induce malignant transformation, and by using MeDIP-sequence analysis, it is found that the methylation levels of Wnt2b and Wnt5b genes in the two cells were significantly different. Wnt2b and Wnt5b are key players in the Wnt/β-Catenin signaling pathway (Klaus and Birchmeier, 2008), which has been highly conserved in evolution and is known to control cell growth, differentiation, apoptosis, and self-renewal. This pathway is activated by binding of Wnt ligands to receptors, which increases the stability of β-catenin in the cytoplasm and promotes its translocation to the nucleus, where it modulates the expression of target genes that lead to tumorigenesis (Klaus and Birchmeier, 2008). Studies have shown that this pathway is abnormally activated during the development of lung cancer and may coordinate or antagonize other signaling pathways to regulate proliferation, migration, and invasion in lung cancer (Reya and Clevers, 2005; Berndt and Moon, 2013). Recently, 30–40% of cells in tumor tissues have been shown to express Wnt ligands, which create a microenvironment that is suitable for tumor cells. In a human lung adenocarcinoma model, 70% of cells have abnormal activation of the Wnt pathway, and 80% of cells may be involved in the formation of the tumor microenvironment, which is critical for the progression of lung cancer (Tammela et al., 2017).

Given the decisive role of the Wnt signaling pathway in the development of lung cancer, inhibition of Wnt ligands provides a viable approach for reducing the expansion of lung cancer cell lines. The purpose of this study was to investigate whether PARG gene silencing can inhibit lung cancer development induced by BaP and whether it can regulate the Wnt ligands to inhibit the development of lung cancer. On the basis of our findings, PARG gene and Wnt ligands may constitute a new option for the diagnosis and treatment of lung cancer.

## Materials and Methods

### Materials

BaP (CAS50-32-8, purity ≥96%) was purchased from American Sigma Company, and dissolved in dimethylsulfoxide (DMSO). Other chemicals were purchased from Sigma–Aldrich (St Louis, MO, United States) or Thermo Fisher Scientific (Shanghai, China), unless otherwise stated.

### Animals and Treatment

The PARG knockout mice [B6N (Cg)-Parg^tm2b(KOMP)Mbp^/J]were purchased from the Jackson Laboratory, and WT mice (C57BL/6J) were purchased from Guangdong Medical Lab Animal Center. PARG knockout mice were generated by the targeted mutation 2b of the Parg gene resulting in deletion of the full-length isoform of PARG protein (PARG_110_). The strategy of gene targeting is Cre-mediated excision of the parental Parg^tm2b(KOMP)Mbp^ allele resulted in the removal of the promoter-driven neomycin selection cassette and critical exon(s) leaving behind the inserted lacZ reporter sequence. We screened for heterozygous PARG knockout mice (PARG^+/−^) in our study since death of homozygous PARG knockout mice (PARG^−/−^) occurring before the normal life span of an organism, occurring during pregnancy, parturition or lactation. The mice were maintained under semi-specific-pathogen-free conditions with the temperature controlled at 23 ± 2°C and a 12-h light/dark cycle. We selected 2-month-old PARG^+/−^ and WT mice for this study. The mice were randomly divided into two groups with 6 per group referring to the principles of experimental animal selection and references. And then, they were treated with 0 or 10 μg/m^3^ aerosols through respiratory tract by a dynamic inhalation cabinet (Jiufang Company, Guangzhou) for 90 or 180 days. The dynamic inhalation device makes liquids into aerosols with a diameter of only a few micrometers, which is in line with the actual human exposure to BaP in the air. At the end of the experiment, mice were anesthetized with ether and blood was collected by eyeball sampling. The mice were then euthanized and the lungs were excised rapidly. Half of each lung was stored in 4% paraformaldehyde, and the other half was stored at -80°C. All animal experiments and procedures were approved by the Shenzhen Center for Disease Control and Prevention. Efforts were made to minimize animal suffering and reduce the number of mice used in the experiments.

### Genotyping of PARG Knockout Mice

Genomic DNA was purified from mouse tails using TianAMP genomic DNA kits (Tiangen, Beijing, China). The concentration and the quality of DNA were assessed by ultraviolet (UV) absorbance using a NanoDrop ND-2000 spectrophotometer (Thermo Fisher Scientific). The DNA was then amplified by PCR (94°C for 2 min; 10 cycles of 94°C for 20 s, 65°C for 15 s, and 68°C for 10 s; 10 cycles of 94°C for 15 s, 60°C for 15 s, and 72°C for 10 s; 72°C for 2 min, 10°C hold) using primers provided by the Jackson Laboratory (Wild-type Forward: 5′-GAG ATA TCT AAG TCA GAG AAA GGT GGT-3′, Wild-type Reverse: 5′-CCT CCT CTG GTG TGT CTG AAG-3′, Mutant Forward: 5′-CGG TCG CTA CCA TTA CCA GT-3′, Mutant Reverse: 5′-GGT ATC AGC GAT GGT TGT TC-3′). The PCR products were 279 bp for the WT sample, and 279 and 507 bp for the heterozygous PARG knockout (PARG^+/−^) sample.

### Hematoxylin and Eosin Staining

Mouse lung tissues were fixed in 4% paraformaldehyde for 48 h, dehydrated in ethanol and embedded in paraffin by using a TissueWave^TM^ 2 Microwave Processor (Thermo Fisher Scientific). Paraffin-fixed tissues were sliced into 5 μm sections, mounted on glass slides, and dried for 1 h. After dewaxing and rehydration, sections were stained with hematoxylin and eosin (Sigma-Aldrich) and examined by light microscopy. The pathology was evaluated by a blinded observer to detect the degree of malignancy.

### Real-Time Quantitative PCR

Total RNA was extracted from frozen lung samples with miRNeasy mini kits (Qiagen, China) according to the manufacturer’s instructions. Complementary DNA (cDNA) was synthesized from 500 ng of total lung RNA (*n* = 3 per group) using the PrimeScript^TM^ RT reagent kit (Takara, China). Quantitative PCR (qPCR) was performed on the ABI Prism 7500 system (Applied Biosystems, Foster City, CA, United States) using SYBR select master mix. The mRNA primers were purchased from Sangon Biotech (Shanghai, China) and are listed in Supplementary Table S1. Experiments were repeated at least 3 times. The relative level of mRNA for each gene was determined using the 2^−ΔΔCt^ method (Schmittgen and Livak, 2008), and *P*-values were calculated using the Student’s *t*-test on replicate 2^−ΔCt^ values for each gene in each treatment group compared to the control group.

### Immunohistochemistry

Mouse lung tissues were fixed in 4% paraformaldehyde for 48 h, dehydrated in ethanol and embedded in paraffin by using a TissueWave^TM^ Microwave Processor (Thermo Fisher Scientific). After dewaxing and rehydration, 5 μm-thick coronal sections were incubated in 0.01 M citrate buffer (pH 6.0) with 0.1% Tween-20 at 95–100°C for 10 min for antigen retrieval. For immunochemistry of Wnt2b and Wnt5b (*n* = 3 per group), the sections were incubated at 4°C overnight with primary antibody (Wnt2b at 1:200 or Wnt5b at 1:50). After being washed with PBST, the sections were stained using the mouse and rabbit-specific HRP/DAB (ABC) detection IHC kit (Abcam, ab64264) and analyzed using an Olympus BX60 compound microscope (Tokyo, Japan).

### Western Blot Analysis

Lung proteins (*n* = 3 per group) were extracted from 30 mg lung tissue with 600 μL lysis buffer (Beyotime, China) and 6 μL protease and phosphatase inhibitor cocktail (Thermo Fisher Scientific, United States) on ice, and then centrifuged and collected. The protein concentration was measured with a BCA protein assay kit (Thermo Fisher Scientific, United States). Each protein sample was combined with loading buffer and heated for 8 min at 100°C. Protein samples were separated on 10% PAGE gels with 5% stacking gels and transferred to PVDF membranes. The membranes were incubated in TBST buffer containing 5% milk at room temperature for 2 h. Subsequently, they were incubated with anti-PARG (mouse monoclonal antibody, 1:100), anti-phosphotyrosine (PY20, mouse monoclonal antibody,1:1000), anti-Wnt2b (rabbit monoclonal antibody, 1:3000), anti-Wnt5b (mouse monoclonal antibody,1:500), or anti-α-tubulin (mouse monoclonal antibody, 1:3000) in TBST buffer for 1.5 h at room temperature. After washing with TBST three times, the membranes were incubated with homologous secondary antibody (anti-rabbit or anti-mouse IgG HRPs) in TBST buffer for 60 min. The membranes were then repeatedly washed with TBST buffer, developed using chemiluminescence reagents from an ECL kit (Pierce ECL, Santa Cruz, CA, United States) and detected on a phosphorimager. The images of the membranes were analyzed by ImageJ software.

### Statistical Analysis

The histograms and statistical analyses of the relative expression of each group were completed using Graph-Pad prism 7.0 software (GraphPad Software, Inc.). Data are presented as mean ± SD. Comparisons between two groups were conducted with the Student’s *t*-test. *P* < 0.05 was considered statistically significant.

## Results

### Genotyping of PARG Knockout Mice

**FIGURE 1:**
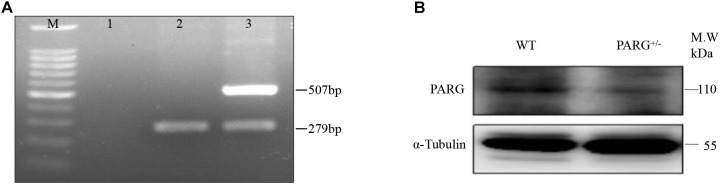
Genotyping of poly (ADP-Ribose) glycohydrolase (PARG) knockout mice. Genotyping of PARG^+/−^ mice. **(A)** Genotyping by PCR. Lane M, 100 bp DNA Marker; Lane 1, blank control; Lane 2, WT mice; and Lane 3, PARG^+/−^ mice. **(B)** Genotyping by Western blotting. The expression of PARG_110_ protein was assessed in lungs from WT and PARG^+/−^ mice.

The heterozygous PARG knockout mice were used to characterize the role of PARG in protecting mice from BaP-induced lung cancer. According to the law of Mendelian inheritance, the genotype of the progeny mice may be WT (PARG^+/+^), heterozygous (PARG^+/−^), or homozygous (PARG^−/−^). Based on genomic DNA purified from mouse tails, PARG^+/−^ mice were screened for our study as PARG^−/−^ mice cannot survive to maturity. The PCR product from WT mice was 279 bp, and the PCR products from PARG knockout heteroygotes (PARG^+/−^) were 279 and 507 bp, as shown in Figure 1A. After BaP exposure, proteins from the lung tissues were extracted and Western blotting were performed to verify the expression of full-length isoform (PARG_110_). As expected, the expression of PARG_110_ was significantly greater in WT mice than in PARG^+/−^ mice (Figure 1B). The results confirm that heterozygous PARG knockout mice were successfully bred in our experiments.

### PARG^+/−^ Mice Are Protected From Pathological Changes in Lung Tissues Induced by BaP

**FIGURE 2:**
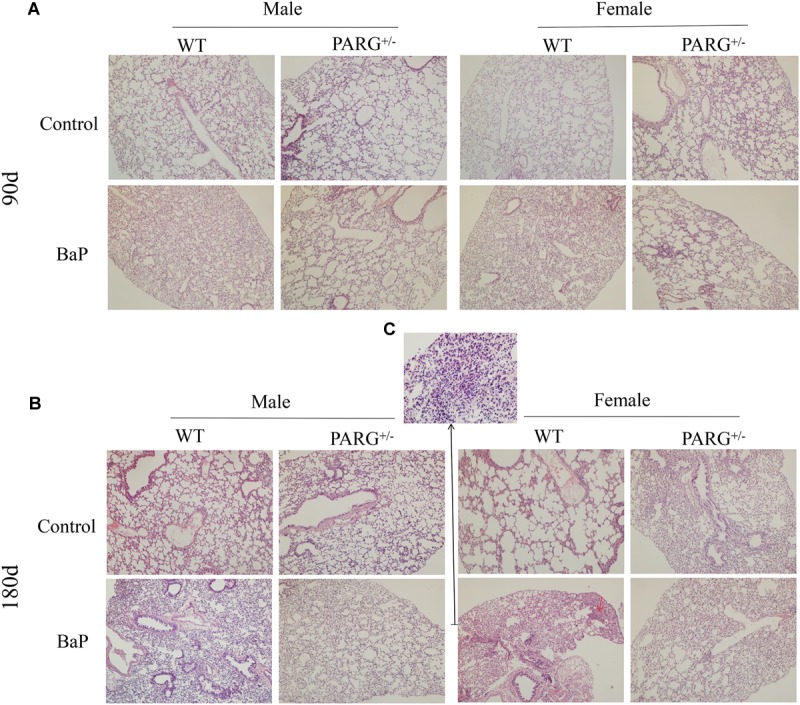
PARG^+/−^ mice are protected from pathological changes in lung tissues induced by BaP inhalation exposure. Pathological changes in lung tissues of WT and PARG^+/−^ mice after benzo(a)pyrene inhalation exposure **(A)** 90-day exposure (×100). **(B)** 180-day exposure to BaP (×100). The red arrows show abnormally increased numbers of cells. **(C)** The magnification of the place pointed by the red arrow in **B**, pathological signs of tumorigenesis (×200). Results are representative of 3 mice from each group.

To establish a lung cancer model for assessing the effects of heterozygous PARG silencing, we exposed mice to long-term inhalation of BaP and then prepared paraffin sections of lung tissues. Hematoxylin and eosin staining were used to analyze the pathological changes that were observed under light microscopy. As shown in Figure 2A, in the lungs of WT mice exposed for 90 days, alveolar diffuse interstitialization occurred, though the alveolar structure was visible; in contrast, the degree of injury in PARG^+/−^ mice was mild with no obvious pathological damage. The results were similar in both male and female mice. After 180-day exposure to BaP, the lungs of the WT mice treated with BaP showed severe alveolar diffuse interstitialization, and the alveolar structure was severely damaged with obvious inflammatory infiltration and abnormal nodules (Figure 2B). Comparison between the 90- and 180-d pathology suggests that the degree of lung injury in WT mice treated with BaP was positively correlated with the time of exposure. In PARG^+/−^ mice after 180 days, however, some alveolar interstitial thickening appeared while the alveolar structure was still visible. This suggests that PARG^+/−^ mice were protected from the effects of BaP on lung pathology. A higher magnification was used to examine tumor formation. In WT mice, the number of cells increased abnormally and tumorigenesis could be observed (Figure 2C); however, no tumor tissue was found in PARG^+/−^ mice. These results demonstrate that heterozygous PARG gene silencing can inhibit the induction of lung cancer by BaP in mice.

### PARG^+/−^ Mice Express Elevated Levels of Phosphorylated Proteins in Lung Tissues After BaP Inhalation Exposure

**FIGURE 3:**
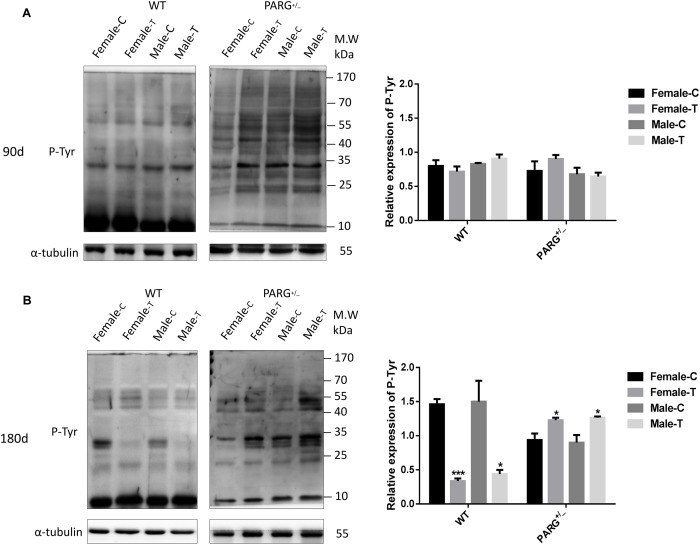
PARG^+/−^ mice express elevated levels of phosphorylated proteins in lung tissues after BaP inhalation exposure. Expression of phosphorylated proteins in lung tissues of mice after BaP inhalation exposure. **(A)** The overall phosphorylation level of proteins in WT and PARG^+/−^ mice that were untreated or were treated with exposure to BaP for 90 days. **(B)** Expression of phosphorylated proteins after 180-day exposure to BaP. Female-C, control untreated female mice; Female-T, treated female mice; Male-C, control untreated male mice; Male-T, treated male mice. ^∗^*P* < 0.05, ^∗∗∗^*P* < 0.001, significant difference in treated compared to untreated mice. Results represent the mean ± SD of 3 mice from each group. Quantification of the phosphorylation levels was performed using ImageJ software.

To determine whether heterozygous PARG silencing affects the overall protein phosphorylation level, we performed Western blot assays using the universal anti-tyrosine phosphorylation monoclonal antibody PY20 with protein extracted from lung tissues. As shown in Figure 3A, the levels of total phosphorylated proteins in WT and heterozygous PARG knockout mice were not significantly different from that of the control group after exposure to BaP for 90 days (*P* > 0.05). After 180-d exposure, however, the level of phosphorylated proteins was significantly down-regulated in WT mice (^∗^*P* < 0.05), but was significantly up-regulated in PARG^+/−^ mice compared with the control group (^∗^*P* < 0.05). These results indicate that, at an extended BaP exposure time, PARG affects phosphorylation of proteins, which could potentially be associated with the ability of PARG**^+/−^** mice to resist tumorigenesis.

### PARG Silencing Inhibits the Relative Expression of Wnt2b and Wnt5b mRNA in Lung Tissues After BaP Inhalation Exposure

**FIGURE 4:**
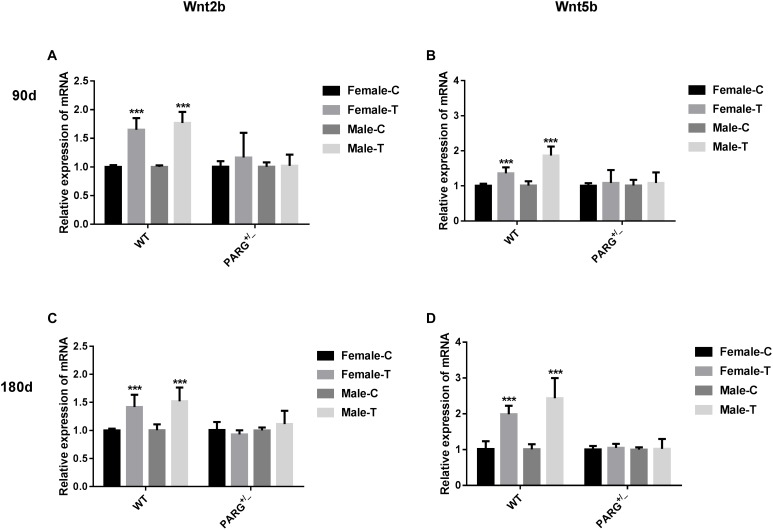
PARG silencing inhibits the relative expression of Wnt2b and Wnt5b mRNA in lung tissues after BaP inhalation exposure. Relative expression of Wnt2b and Wnt5b mRNA in lung tissues of WT and PARG^+/−^ mice after BaP inhalation exposure. The mRNA expression in lungs from WT and PARG^+/−^ mice was measured by real-time quantitative PCR after 90 days (panels **A**,**B**) or 180 days (panels **C**,**D**) of BaP inhalation exposure. Female-C, control untreated female mice; Female-T, treated female mice; Male-C, control untreated male mice; Male-T, treated male mice. ^∗∗∗^*P* < 0.001, significant difference was found in treated compared to untreated mice. Results represent the mean ± SD of 3 mice from each group.

To further elucidate whether ADP-ribosylation affects the Wnt pathway in PARG^+/−^ mice, we first performed real-time qPCR to detect the relative expression of the Wnt2b and Wnt5b genes. The relative expression of Wnt2b and Wnt5b mRNA was significantly higher in WT mice than in control mice at 90 and 180 days (^∗∗∗^*P* < 0.001), but there were no significant differences in the PARG^+/−^ mice (*P* > 0.05) (Figure 4).

### PARG Silencing Inhibits the Expression of Wnt2b and Wnt5b Protein in Lung Tissues After BaP Inhalation Exposure

**FIGURE 5:**
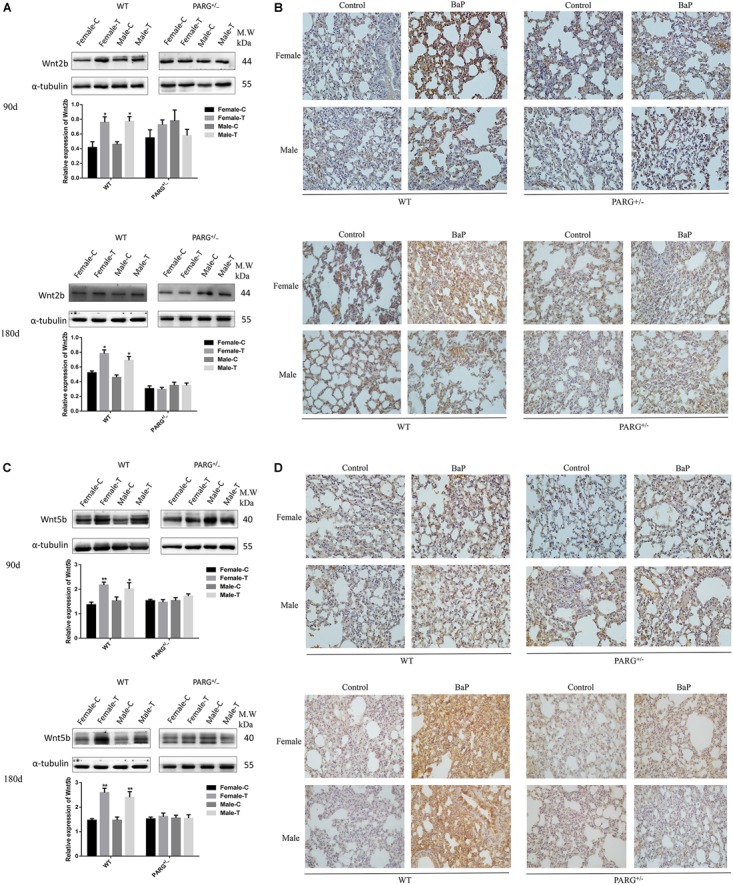
PARG silencing inhibits the expression of Wnt2b and Wnt5b protein in lung tissues after BaP inhalation exposure. Expression of Wnt2b and Wnt5b protein in lung tissues detected by Western blotting and immunohistochemistry. **(A)** Western blotting of Wnt2b expression. **(B)** Immunohistochemical staining of Wnt2b (×200). Protein expression levels are reflected by the area and depth of brownish yellow. **(C)** Western blotting of Wnt5b expression. **(D)** Immunohistochemical staining of Wnt5b (×200). Red arrows indicate Wnt2b and Wnt5b localization in the cytoplasm. Female-C, control untreated female mice; Female-T, treated female mice; Male-C, control untreated male mice; Male-T, treated male mice. ^∗^*P* < 0.05, ^∗∗^*P* < 0.01, significant up-regulation in treated vs. control mice.

The expression of Wnt2b and Wnt5b at the level of the protein were further confirmed by performing Western blotting and immunohistochemistry. The expression of Wnt2b protein was up-regulated in lungs from WT mice that were treated with BaP for 90 and 180 days (^∗^*P* < 0.05, compared with the control group); however, for PARG^+/−^ mice, no statistically significant differences were observed (*P* > 0.05) (Figure 5A). In immunohistochemistry assays, Wnt2b protein (brownish yellow staining) was localized to the cytoplasm, and after 90 and 180 days of BaP inhalation exposure, the expression levels in WT male and female mice were higher for treated vs. control mice; however, for PARG^+/−^ mice, there were no significant differences (Figure 5B). Similar results were observed for Wnt5b, though the effect on Wnt5b expression was more obvious at 180 days than at 90 days (Figure 5C,D). These findings suggest that PARG gene silencing stabilizes the expression of Wnt2b and Wnt5b after BaP exposure, possibly inhibiting the progression of lung cancer.

## Discussion

Metabolically activated BaP is known to cause cytotoxic, teratogenic, genotoxic, mutagenic and carcinogenic effects in many different tissues and cell types from numerous mammalian studies (Miller and Ramos, 2001; van Delft et al., 2010). BaP in cigarette smoking is implicated as one of the main factors in lung cancer (Rubin, 2001). The occurrence of cancer includes three stages: initiation, promoting and progressing. Epigenetic modification, as a bridge between these stages, can involve DNA methylation, microRNA, chromatin remodeling, and histone modification (Bird, 2007). ADP-ribosylation is one of the most important post-translational modifications in tumorigenesis (Klaus and Birchmeier, 2008). Studies showed that the use of PARG inhibitor to suppress PARG activity facilitates oxidative damage-induced PARylation as well as DNA damage repair (Zhang et al., 2015). PARG gene silencing increases the level of poly (ADP-ribosylation) to regulate DNA damage repair and genome stability (Koh et al., 2004). In our previous study, it is determined that *in vitro* PARG silencing inhibits tumorigenesis by dramatically reducing DNA damage, chromosome abnormalities, micronuclei formations, and malignant transformation. To further investigate the possible *in vivo* role of PARG gene silencing, heterozygous PARG knockout mice were utilized. We exposed WT and PARG^+/−^ mice to BaP by dynamic inhalation for 90 and 180 days. Pathological analysis showed that carcinogenesis appeared in the lungs of WT mice and the injury was progressive for 180-day vs. 90-day treatment, while PARG^+/−^ mice showed no carcinogenesis and minimal signs of lung injury. These results suggest that PARG gene silencing can inhibit lung cancer induced by BaP in mice, which is consistent with our *in vitro* results.

In our previous vitro study, we identified two distinct Wnt ligands (Wnt5b and Wnt2b) that are modulated by PARG by using the MeDIP-sequence techniques. This raises the possibility that ADP-ribosylation may affect the carcinogenesis of BaP by regulating the activation of the Wnt signaling pathway after PARG gene silencing. The Wnt pathway consists of three components: the Wnt/β-catenin canonical pathway, the Wnt/Ca^2+^ pathway and the Wnt/polarity pathway (Wodarz and Nusse, 1998). After activation of the canonical pathway, Wnt ligands bind to Frzzled and LRP5/6 on the cell surface to form a trimer, which weakens the stability of a destruction complex composed by β-catenin, Axin, GSK-3β, and APC to prevent the phosphorylated degradation of β-catenin. The concentration of β-catenin increases in the cytoplasm and then is transferred into the nucleus which ultimately activate the expression of downstream target genes (Veeman et al., 2003). During this process, protein phosphorylation, especially tyrosine phosphorylation (P-Tyr), as a major mode of cell signal transduction and regulation of enzyme activity, plays an vital role in the regulation of β-catenin (Ikeda et al., 1998). ADP-ribosylation can promote phosphorylated proteins to bind to Axin scaffolding proteins, affecting the stability of the key protein β-catenin and regulating the activation of the Wnt pathway (Yang et al., 2016). In the current study, the level of total phosphorylated protein in WT mice and PARG^+/−^ mice was not significantly different after 90-day exposure to BaP. However, after 180 d, phosphorylated protein was significantly reduced in WT mice but was up-regulated in PARG^+/−^ mice compared with the control group. These findings are consistent with the possibility that, as the exposure time of BaP extended, loss of PARG promotes phosphorylation of proteins, which possibly leads to phosphorylated degradation of key proteins in the Wnt pathway; supported by the following studies (Zeng et al., 2005; Kim et al., 2013; Yang et al., 2016). We will try to explore how does PARG regulates protein tyrosine phosphorylation to regulate the Wnt signaling against the progression of lung cancer in our next study.

Wnt ligands play a vital role in the development of lung cancer, and inhibition of Wnt ligands may reduce the expansion of lung cancer cell lines (Tammela et al., 2017). Our results demonstrate that the relative expression of Wnt2b and Wnt5b mRNA was up-regulated in lung tissues of WT mice compared with the control group after 90- and 180-day exposure to BaP. Furthermore, the expression of Wnt2b and Wnt5b protein was up-regulated, though there were no significant differences in Wnt2b and Wnt5b mRNA and protein expression in PARG^+/−^ mice. It suggested that loss of PARG stabilized the expression of Wnt ligands, probably suppressing the activation of the Wnt pathway against the progression of lung cancer.

Wnt2b and Wnt5b are two ligands of the Wnt signaling pathway. Wnt2b mainly acts through the canonical Wnt pathway and binds to receptors on the cell membrane to increase the stability of β-catenin in the cytoplasm and promote its translocation to the nucleus to activate downstream target genes that lead to tumorigenesis (Roelink et al., 1992). Studies have shown that Wnt2b is overexpressed in various cancers (Katoh, 2001; Huang et al., 2015). Wnt5b, on the other hand, is a non-canonical Wnt pathway factor that activates the Wnt/Ca^2+^ pathway or blocks the down-regulation of β-catenin by GSK-3β to prevent the classical Wnt pathway (Kohn and Moon, 2005). Studies have shown that Wnt5b plays different roles in different types of cancers. In some cancers, such as lung cancer, it promotes tumorigenesis, and in other cancers, it suppresses tumorigenesis (Kikuchi and Yamamoto, 2008; Harada et al., 2017). On the basis of its different roles in different cancers, Wnt5b may constitute a specific marker for lung cancer screening. In our study, it is found that the up-regulation of Wnt2b was similar at 90 and 180 days, while the up-regulation times of Wnt5b was more obvious at 180 days than at 90 days. These findings may suggest that the Wnt non-canonical pathway increased with extended exposure times, while the classical pathway remains activated at both 90 and 180 days. Specific mechanisms of interaction between the two pathways remains to be further studied.

**FIGURE 6:**
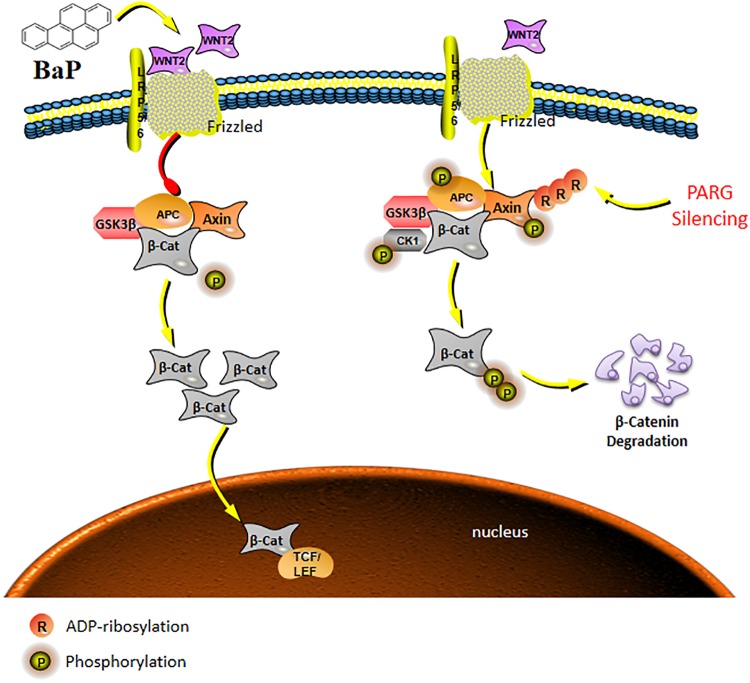
Schematic model of the Wnt/β-catenin signaling pathway regulated by PARG gene silencing during BaP-induced lung cancer. After activation of the canonical pathway, Wnt ligands bind to Frzzled and LRP5/6 on the cell surface to form a trimer, which weakens the stability of a destruction complex composed by β-catenin, Axin, GSK-3β, and APC to prevent the phosphorylated degradation of β-catenin. The concentration of β-catenin increases in the cytoplasm, and then transfers into the nucleus to ultimately activate the expression of downstream target genes. PARG gene silencing may promote binding of phosphorylated proteins to the Axin scaffolding proteins, affecting the stability of the key protein β-catenin, and then suppressing the activation of the Wnt/β-catenin pathway to stabilize the expression of Wnt2b against the progression of lung cancer.

In conclusion, in the development of lung cancer induced by BaP, the expression of Wnt ligands are up-regulated, which is consistent with current understanding of the role of this pathway. Additionally, PARG gene silencing may regulate the phosphorylation level of proteins to stabilize the expression of Wnt2b, possibly inhibiting the ability of Wnt/β-catenin pathway to drive lung cancer progression as shown in the schematic model in Figure 6. The mechanism how PARG gene silencing affects the expression of Wnt5 remains to be futher explored. Understanding of the unresolved issue will contribute to the development of applications of PARG for cancer therapy. Lung cancer is one of the world’s most serious threats to human health and has become a global public health problem (Siegel et al., 2018). Therefore, studying the mechanisms of lung cancer provides increased understanding that is relevant to its diagnosis and treatment. Though epigenetic modification is extensive, basic and reversible, its theory and results are gradually being applied to the diagnosis and treatment of cancer (Dawson and Kouzarides, 2012). In this study, it is shown that PARG gene silencing can prevent the occurrence of lung cancer induced by BaP. Our results demonstrate that PARG may be a target for the diagnosis and treatment of lung cancer. Furthermore, the inhibition of Wnt ligands may inhibit lung cancer. These results provide a new potential approach for the treatment of lung cancer. In concludsion, the use of Wnt ligands in the diagnosis of lung cancer and the use of PARG inhibitors as a potential therapeutic against lung cancer is supported.

## Ethics Statement

This study was carried out in accordance with the Principles of Laboratory Animal Care (NIH publication No. 80–23, revised 1985). The protocol was approved by the Regulations for Animal Care and Use Committee of Experimental Animal Center at Shenzhen University.

## Author Contributions

HH, WD, and JL conceived the project. WD, YF, and YD performed the experiments. WD, YD, DW, XL, LY, and KL analyzed the results. WD, ZZ, PG, HL, JZ, and XX wrote the first draft. All authors revised the manuscript. HH and GH edited and approved the manuscript.

## Conflict of Interest Statement

The authors declare that the research was conducted in the absence of any commercial or financial relationships that could be construed as a potential conflict of interest.

## Supplementary Material

TABLE S1 Primers used for real-time quantitative PCR.
