# Supplementary material for: Regulation of Wnt Singaling Pathway by Poly (ADP-Ribose) Glycohydrolase (PARG) Silencing Suppresses Lung Cancer in Mice Induced by Benzo(a)pyrene Inhalation Exposure
Source: Front Pharmacol. 2019 May 3;10:338. doi: 10.3389/fphar.2019.00338 (PMC6509174; doi:10.3389/fphar.2019.00338)
Supplement: TABLE S1 — Primers used for real-time quantitative PCR. [file Table_1.DOCX]

**Table 1**

| Mice gene | Oligonucleotide sequence | Product size |
| --- | --- | --- |
| Wnt2b | F: 5’-CCT TGG GAC TTC CCC CAC AT-3’ | 162bp |
|  | R: 5’-AAG CTG AAG ATC CTC AAG AAC TAG C-3’ |  |
| Wnt5b | F: 5’-CTG CTG ACT GAC GCC AAC T-3’ | 145bp |
|  | R: 5’-CCT GAT ACA ACT GAC ACA GCT TT-3’ |  |
| GAPDH | F: 5’-GGTTGTCTCCTGCGACTTCA-3’ | 183bp |
|  | R: 5’-TGGTCCAGGGTTTCTTACTCC-3’ |  |
